# Risk and treatment of symptomatic epidural hematoma after anterior cervical spine surgery: A retrospective clinical study

**DOI:** 10.1097/MD.0000000000018711

**Published:** 2020-01-10

**Authors:** Yehui Liao, Yang Tian, Rupei Ye, Chao Tang, Qiang Tang, Fei Ma, Sheng Yang, Hongchun He, Dejun Zhong

**Affiliations:** a Department of Spinal Surgery; b Department of Pathology, The affiliated Hospital of Southwest Medical University, Luzhou city, Sichuan province, China.

**Keywords:** cervical spinal surgery, incidence, risk factor, spinal epidural hematoma, treatment

## Abstract

Symptomatic epidural hematoma (SEH) after anterior cervical spine surgery is very rare, but it has disastrous consequences for the patients. Timely diagnosis and evaluation can effectively reduce the sequelae of neurological deficit in SEH. The purpose of this study was to retrospectively analyze a subset of clinical data of SEH after anterior cervical spine surgery, and to investigate the risk factors and treatment experience of this serious complication.

Neurological deterioration after anterior cervical spine surgery was detected in six patients. Epidural hematoma was confirmed by emergency cervical magnetic resonance imaging (MRI). The patients included five males and one female, with an average age of 56.7 ± 13.1 years (range 42–76 years). Three patients had a history of drinking and/or smoking. All of the patients were treated with nonsteroidal anti-inflammatory drugs (NSAIDs) preoperatively, but without anticoagulant drugs or pre-spinal surgery. The coagulation function was normal in all patients. Except for one patient, who had lower blood pressure (BP) during the operation and higher BP after the operation, the other patients had a normal level of BP during the pre-, intra-, and post-operation periods. The average time was 9.9 ± 6.7 hours (range, 2−19 hours) from the postoperative period to the initial neurological deficit and 6.3 ± 6.0 hours (range, 1.8−16.7 hours) from the initial deterioration to evacuation. Five patients underwent emergency evacuation, and one patient underwent conservative treatment. Four patients who underwent evacuation and one patient who received conservative treatment achieved neurological function recovery with an American Spinal Injury Association (ASIA) grade 2.4 ± 0.9 (range, 2−4 score) score at the last follow-up. One patient with confirmed arterial epidural hemorrhage during the evaluation showed no neurological function recovery at the last follow-up.

Wide exposure of the epidural space and BP level during the perioperative period play an important role in the formation of SEH after anterior cervical spine surgery. Arterial epidural hematoma has serious consequences; therefore, early diagnosis and evaluation play an important role in the recovery from paralysis.

## 1 Introduction

Since Jackson^[1]^ first reported a case of epidural hematoma in 1869, more than 350 cases of epidural hematoma have been reported in the literature.^[2,3]^ Most patients present with confirmed epidural hematoma after spinal surgery, while a few patients present with a neurological deficit and only show a hematoma on computed tomography (CT) or MRI.^[4,5]^ SEH is considered a source of neurological deterioration after spine surgery, which requires further surgery.^[2]^ SEH after spinal surgery is rare, and its reported incidence is 0.1% to 0.41% in the literature.^[2,6–8]^ However, the complications are very serious and often lead to a neurological deficit,^[9]^ especially for cervical surgery, which can lead to paralysis and even death.^[10,11]^

In a few studies the presence of SEH after anterior cervical surgery was reported, and the influencing factors and treatment strategies were analyzed.^[12–14]^ An analysis of the results shows some limitations,^[8–11]^ such as low incidence and few cases. This study reviewed six cases with clinical data of SEH following cervical diseases (cervical spondylotic radiculopathy (CSR) and cervical spondylotic myelopathy (CSM)) in the past 10 years, and it aimed to provide more possibilities for the etiology of SEH, more options for the treatment of such complications, and more options for the prevention of such diseases.

## 2 Methods

All medical history data collected in this article were obtained from the patients and confirmed by them. This study was approved by the Ethics Committee of the Affiliated Hospital of Southwest Medical University (KY2019147). From January 2009 to December 2018, we treated 6890 patients with CSR and CSM. Neurological deterioration was observed after the operation, and epidural hematomas were confirmed by cervical MRI in 6 (0.09%) cases, including 5 males and 1 female, with an average age of 56.7 ± 13.1 years (range, 42−76 years). We retrospectively analyzed the history, drug treatment, BP during the pre-, intra-, and postoperative periods, hematoma evacuation surgery, complications, and neurological function of these 6 patients. Neurological function was assessed by the ASIA grade.

## 3 Results

Table 1 shows the diagnosis of patients during the initial preoperative evaluation, previous medical history, coagulation function, and drug treatment. The main diagnoses in the 6 patients were CSR and CSM. Three patients had a history of drinking, and the amount of alcohol consumed per week was less than 10 units; in 1 case, this amount was greater than 10 units per week. Three patients had a history of smoking; one patient smoked about 20 cigarettes a day, one patient smoked less than 10 cigarettes a day, and one patient had quit smoking 3 years before surgery. All patients were treated with NSAIDs preoperatively, and the average treatment duration of 11.5 ± 5.1 days (range, 7−20 days). No anticoagulant drugs and pre-spinal surgery were performed. Activated Partial Thromboplastin Time (APTT), Prothrombin Time (PT), International Normalized Ratio (INR), Platelet, Hemoglobin (Hb), and other tests were normal during the preoperative evaluation.

**Table 1:**
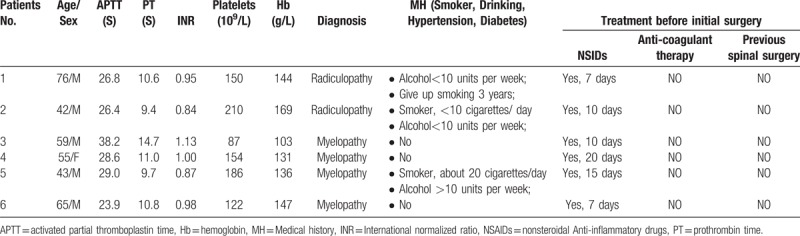
Basic statistics before initial operation.

Table 2 shows the patients’ initial surgical information, treatment strategy, and prognosis. The BP (113−136) / (72−88) mmHg was within the normal range in 6 patients before surgery. Except for patient 4 with a lower BP (73/55 mmHg) during the intra-operative period and higher BP (160/88 mmHg) during the postoperative period, the other 5 patients had a normal BP during the intra-operative and postoperative periods. The average blood loss was 163.3 ± 117.8 ml (range, 50−380 ml) during the operation, and a drainage tube was placed regularly after the operation in 6 patients. The average time from the postoperative period to the initial neurological deficit was 9.9 ± 6.7 hours (range, 2−19 hours). MRI confirmed the presence of a hematoma in patient 2, but evacuation was not performed because of the gradual recovery of paralysis at about 2 hours after the initial neurological deterioration. Evacuation was immediately scheduled in the other 5 patients, when MRI confirmed an epidural hematoma. The mean time from initial deterioration to evacuation was 6.3 ± 6.0 hours (range, 1.8−16.7 hours). An epidural hematoma was confirmed during the evacuation, and 1 patient also had confirmed arterial epidural bleeding. The average follow-up period was 20 ± 10.5 months (range, 6−36 months). Except for patient 4, who did not achieve neurological function recovery, the other 5 patients had 2.4 ± 0.9 (range, 2−4 score) score improvement by the ASIA grade at the follow-up time.

**Table 2:**
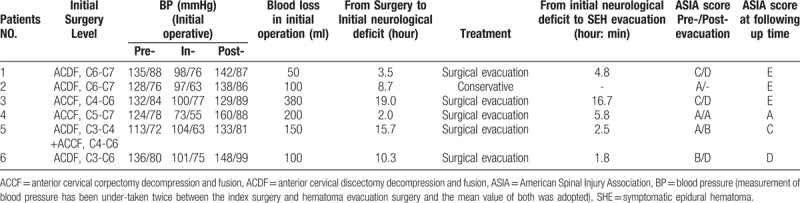
Initial surgery information, treatment strategy, and following.

## 4 Case reports

### 4.1 Case 1

**Figure 1:**
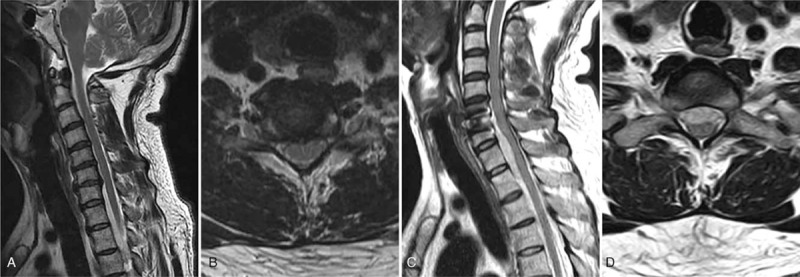
A 42-year-old man presented with CSR (case 1). (A, B) Sagittal and axial MRI before the initial operation showed cervical degeneration, and the C6/7 intervertebral disc was protruding on the right side; (C, D) Sagittal and axial MRI showed epidural hematoma at the C6-thoracic2 (T2) level behind the vertebrae after the ACDF surgery.

Patient 2, male, 42-year-old, was admitted to the hospital for radiation pain and numbness in the right upper extremity since the last 2 years. After obtaining the medical history, and performing comprehensive physical and imaging examinations, shown in Figure 1 and Table 1, the patient was diagnosed with CSR, and C6-7 ACDF was performed. During the operation, the C6-7 disk and epiphysis on the post-downward margin of C6 and the post-upper edge of C7 were resected. After stopping the bleeding and placing a cage, a titanium plate, and a drainage tube, the incision was sutured. BP was maintained at a mean level of 97/63 mmHg during the intra-operative period. After recovering from anesthesia, the patient's symptom was significantly relieved, and the movement of limbs was normal.

At 8.7 hours after surgery, the patient's sensation disappeared below the level of C6 and perianal and saddle areas, muscle strength was 0 grade, ASIA grade was grade A, tendon reflexes disappeared, and pathological signs were negative. The drainage tube was unobstructed, and it drained 10 ml of bloody fluid. Mannitol and prednisolone were used, and cervical MRI examination was planned immediately. Epidural hematoma was detected at the C5-thoracic 2 (T2) level behind the vertebrae on the MRI image (Fig. 1). Fortunately, the patient gradually recovered from paralysis at about 2 hours after the initial neurological deficits. Muscle strength recovered to a 2−3 score, ASIA grade recovered to grade C, the reflex disappeared, and pathological signs were negative. Therefore, evacuation was not performed, and mannitol and prednisolone were used. At about 6 hours after the initial neurological deficit, muscle strength of the limbs recovered to a 3−4 score, sensation over the perianal and saddle areas was diminished, and ASIA grade returned to grade D. ASIA grade returned to grade E at 24 hours postoperatively. The neurological deficit was not aggravated after 1 year of follow-up.

### 4.2 Case 2

Patient 4, female, 55-year-old, was admitted to our department because of numbness of limbs, unstable gait, and cotton wool feeling since the last 8 months. She was diagnosed with CSM and ASIA grade D, and she underwent the C5-7 Anterior Cervical Corpectomy Decompression and Fusion (ACCF) surgery under general anesthesia. The mean BP level was about 73/55 mmHg, and no bleeding was detected during the operation. After the patient recovered from anesthesia, limb numbness was relieved significantly when compared with the preoperative level. The muscle strength of the limbs was 4/5 and the ASIA grade was grade D. The patient returned safely to the ward. Her BP was 160/88 mmHg post-operation.

**Figure 2:**
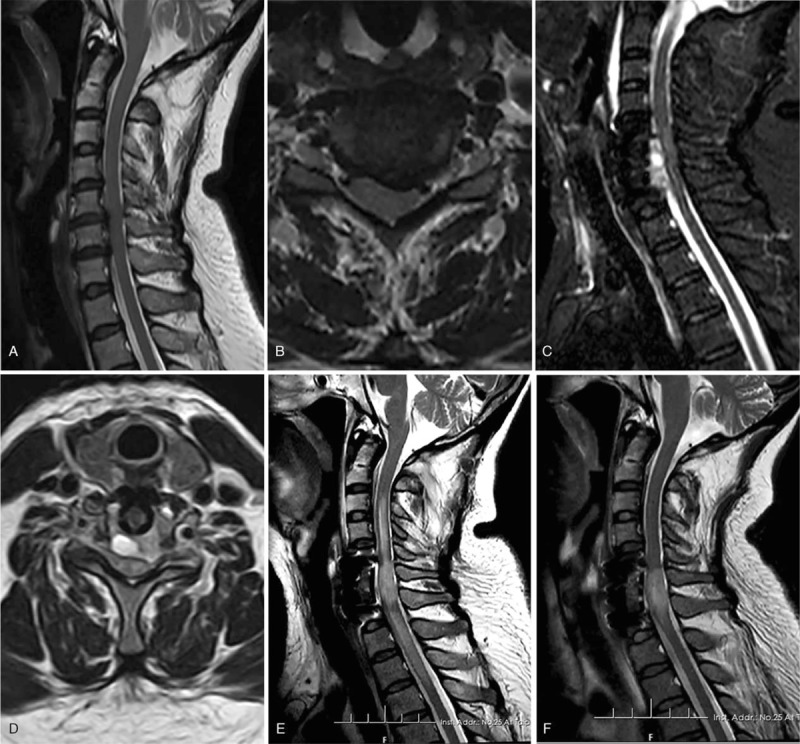
A 55-year-old female presented with CSM (case 2). (A, B) Sagittal and axial MRI before the initial operation showed cervical degeneration, disc herniation, spinal stenosis, and spinal cord compression at the C5/6 and C6/7 levels; (C, D) Sagittal and axial MRI showed epidural hematoma, which severely compressed the cord at the C5−C7 spinal canal level after the ACDF surgery. (E) Epidural hematoma was removed, and spinal cord edema was detected on MRI after evaluation; (F) Cervical MRI showed spinal cord necrosis at the C6−C7 level at 1-month post-evaluation.

Two hours after the operation, the patient again complained of numbness and weakness in her limbs. Physical examination showed sensory disturbance of the extremities, key muscle strength of limbs 3/4, and ASIA grade D. Limb tendon reflexes were weak and the pathological signs were negative. The drainage tube was in its normal position and unobstructed, and it drained 20 ml of bloody fluid. Considering the possibility of hysteria, mannitol and prednisolone were given immediately. Seven hours after surgery, paralysis was not relieved but it had aggravated, and ASIA grade decreased to grade A. an emergency cervical MRI showed an epidural hematoma at the C5−C7 spinal canal level (Fig. 2). At 7.8 hours after surgery, the patient underwent anterior cervical hematoma evacuation. During the operation, a large hematoma around the cage and epidural space was seen, and it compressed the dura mater severely. After removal of the hematoma in the epidural space, arterial bleeding was found in the epidural space near the right posterior location of the C6 corpectomy decompression. After coagulation and confirming that there was no active bleeding, the cage support was fixed with a titanium plate. Then the incision was closed. The mean BP level was 100/80 mmHg. After anesthesia, the sensation and activity of the upper and lower limbs were still absent, the muscle strength was 0, and the ASIA score was grade A. Drug and rehabilitation treatments were performed after evaluation. Cervical MRI showed spinal cord necrosis at the C6-T1 level at 1-week post-evaluation (Fig. 2). No recovery was detected during the 6 months of follow-up time.

## 5 Discussion

Amir^[2]^ reported that multi-segmental surgery is an independent risk factor for postoperative hematoma (*P* = .002), and all patients with epidural hematoma had received more than one disc-segment surgery, which was similar to that suggested by other authors.^[12,15]^ Extensive anterior or posterior segmental cervical surgery increased the damage to the epidural venous plexus, which may be the main cause of intraspinal venous plexus injury.^[2,13]^ The wall of the intraspinal venous plexus is weak, and there is no venous valve in the lumen. Bleeding after injury does not stop spontaneously.^[16]^ Henderson^[17]^ showed that the pressure of the intraspinal venous plexus is lower than that of the cerebrospinal fluid. When the pressure of the hematoma, which was caused by intraspinal venous plexus bleeding, approaches or reaches the same pressure as that of the cerebrospinal fluid, it can compress the wall of the intraspinal venous plexus to achieve hemostasis. In our medical records, two patients with anterior cervical single-segment ACDF developed paralysis postoperatively and MRI confirmed the presence of an epidural hematoma. Paralysis of these two single-segment ACDF patients had recovered, one with evaluation and the other without evaluation. The reasons for this occurrence may be that single-segment ACDF surgery causes less trauma, small exposure, and milder intraspinal venous plexus injury. The four patients treated with ACCF all had large exposure of the epidural space and extensive intraspinal venous plexus injury, which were one of the most important reasons for postoperative hematoma. As the pressure exerted by the hematoma increases up to the level of cerebrospinal fluid pressure, the venous plexus hemorrhage stops automatically.

Hypertension is an important risk factor for postoperative hematoma, but none of reported studies has elaborated the possible mechanism by which BP causes epidural hematoma. Some authors^[18]^ believe that compression of the spinal cord is mainly caused by arterial bleeding in the spinal canal. Guodong^[11]^ believes that arterial hematoma in the spinal canal can be relatively low, because arterial bleeding can be easily detected during surgery. According to our medical records, none of our 6 patients had a history of hypertension before surgery. In patient 4, arterial bleeding was found in the epidural space. No active arterial or venous bleeding was found during the initial operation. We speculated and analyzed the following possible causes: the BP was low in the initial intraoperative period (mean 73/55 mmHg); therefore, small arterial hemorrhage in the epidural space was not detected and bipolar coagulation was not adequate. When the patient recovered from anesthesia, BP (160/88 mmHg) increased and the small arteries that do not achieve adequate hemostasis started to bleed again, resulting in the formation of epidural hematoma. Therefore, we conclude that a hematoma caused by epidural arterial hemorrhage will lead to severe cord injury, and the recovery from paralysis after evacuation will also be very poor, and a hematoma caused by epidural venous plexus hemorrhage will also lead to cord injury, but paralysis may recover by at least grade 1 (ASIA score) after the evaluation.

This study is based on retrospective analysis of medical records. There is a lack of adequate evidence and literature reports to confirm the correctness of our speculation, but we still recommend that the BP should be strictly controlled during the intra- and post-operative periods for ACDF or ACCF. During the in-operation period, the systolic blood pressure (SBP) should not be lower than 90 mmHg and the diastolic blood pressure (DBP) should not be below 60 mmHg to accurately observe the presence of active bleeding in the decompression area. Postoperative BP control (SBP ≤140 mmHg, DBP ≤90 mmHg) also reduced the risk of plexus bleeding.

The other etiologies, such as age more than 60 years, NSAIDs, anticoagulant drugs, excessive bleeding (>1000 ml), drinking (1 week > 10 units), and smoking rate, may be the risk factors for SEH.^[5,11,15,19]^ In this study, average patient age (56.7 ± 13.1 years) and mean blood loss (163.3 ± 117.8 ml) were less than those in the literature reports. All of the six patients were irregularly treated with NSAIDs, but they were not treated with anticoagulation before the operation. Therefore, we do not have sufficient evidence to prove that SEH is related to age, coagulopathy, NSAIDs, anticoagulation, and excessive bleeding. Three patients (50%) in this group had a history of smoking. Two of them had lower levels of smoking, and 1 patient had quit smoking 3 years before the operation. Three patients (50%) had a history of alcohol consumption, and only one patient fulfilled Amir^[2]^ criterion of 10 units of alcohol consumption per week. Although we do not have enough evidence to confirm the relationship between SEH and smoking or drinking, we still need to pay attention to smoking and drinking, which may be one of the risk factors for SEH after cervical surgery in the literature.

Timely diagnosis and treatment can significantly increase the chances of recovery from paralysis,^[8]^ especially for epidural arterial hemorrhage. Patients who underwent evaluation within 24 hours showed significant recovery from paralysis when compared with the preoperative level, and there was no chance of recovery from paralysis if the evaluation was performed after more than 24 hours.^[11,20,21]^ In our group, 5 patients underwent evaluation within 12 hours, and 4 patients showed recovery from paralysis of at least two grades at the last follow-up. The results were satisfactory. In patient 4, paralysis lasted up to the last follow-up. The main reason for this occurrence was that hysteria was initially considered when the patient showed neurological symptoms. Cervical MRI examination was not arranged in a timely manner, and all of these methods failed to diagnose the epidural hematoma earlier and accurately. Therefore, we believe that timely cervical MRI is necessary when there is re-deterioration of neurological function or rapid progress of paralysis after anterior cervical surgery. The hematoma caused by epidural arterial hemorrhage develops rapidly and causes serious damage to the spinal cord. Earlier diagnosis and evaluation play an important role in the recovery from paralysis.

## Acknowledgments

We thank LetPub (www.letpub.com) for its linguistic assistance during the preparation of this manuscript.

## Author contributions

**Data curation**: Yehui Liao, Yang Tian.

**Formal analysis**: Yehui Liao.

**Investigation**: Chao Tang, Qiang Tang, Rupei Ye.

**Methodology**: Dejun Zhong, Fei Ma.

**Project administration**: Dejun Zhong, Yehui Liao.

**Resources**: Sheng Yang, Hongchun He.

**Software**: Chao Tang.

**Supervision**: Dejun Zhong.

**Validation**: Dejun Zhong, Yehui Liao.

**Visualization**: Sheng Yang, Chao Tang.

**Writing – original draft**: Yehui Liao, Yang Tian.

**Writing – review & editing**: Yehui Liao, Dejun Zhong.
